# Effects of Wuqinxi on blood pressure and lipids in hypertensive middle-aged and elderly adults: a meta-analysis

**DOI:** 10.3389/fmed.2026.1929567

**Published:** 2026-09-01

**Authors:** Tianhao Zhang, Mingang Guo, Tao Li, Zehui Zhang

**Affiliations:** School of Physical Education, Wuhan University of Technology, Wuhan, Hubei, China

**Keywords:** health, hypertension, meta-analysis, middle-aged and elderly, Wuqinxi

## Abstract

**Purpose:**

This study aimed to quantify the therapeutic effects of standardized Wuqinxi exercise on blood pressure, lipid profile, and resting heart rate in middle-aged and older adults with primary hypertension, and to grade the evidence certainty using the GRADE framework.

**Methods:**

We systematically searched Web of Science, Wanfang Data, CNKI, EBSCO, and PubMed (January 2000 to June 30, 2026) for randomized controlled trials (RCTs) evaluating Wuqinxi as an adjunctive intervention for hypertension. Eleven RCTs (770 participants, aged 30–80 years, 3–6 months duration) were included. Primary outcomes were changes in systolic blood pressure (SBP), diastolic blood pressure (DBP), high-density lipoprotein (HDL), and low-density lipoprotein (LDL). Meta-analyses were performed using random-effects models, with pre-specified sensitivity analyses excluding studies with high risk of bias, non-standard protocols, or incompatible controls.

**Results:**

Wuqinxi significantly reduced SBP by 5.3 mmHg and DBP by 6.7 mmHg, improved HDL [standardized mean difference (SMD) = 0.71, 95% confidence interval (CI): 0.40–1.02], and reduced LDL (SMD = −0.63, 95% CI: −1.19 to −0.07). GRADE evidence quality was moderate for blood pressure, low for HDL, and very low for LDL.

**Conclusion:**

Wuqinxi is a safe, low-cost adjunctive therapy for hypertension with consistent blood pressure-lowering effects over 3–6 months. We recommend integrating 5–6 sessions weekly into community and clinical hypertension care. Lipid benefits require confirmation through high-quality RCTs with standardized protocols.

**Systematic review registration:**

PROSPERO, identifier CRD420261396941.

## 1 Introduction

Hypertension remains the leading modifiable risk factor for cardiovascular morbidity and mortality globally, affecting more than 1.4 billion adults and contributing to approximately 10.8 million deaths annually (1). Despite advances in pharmacotherapy, global hypertension control rates remain below 50%, with particularly poor outcomes in low- and middle-income countries and aging populations (2). In adults aged 65 years and older, age-related arterial stiffening leads to isolated systolic hypertension in up to 60% of cases, while middle-aged adults often present with combined systolic-diastolic hypertension and concurrent dyslipidemia, further increasing cardiovascular risk (3, 4).

The coexistence of hypertension and dyslipidemia accelerates atherosclerotic progression, leading to target organ damage in the heart, brain, and kidneys, and imposes a substantial economic burden on healthcare systems worldwide (5).

While pharmacotherapy remains the mainstay of hypertension management, challenges such as medication non-adherence, adverse effects, and residual cardiovascular risk have driven urgent demand for safe, accessible, and culturally acceptable non-pharmacological adjuncts (6). Traditional Chinese mind-body exercises have emerged as promising interventions for chronic disease management, with Wuqinxi (Five-Animal Frolics) standing out for its unique dynamic movement characteristics. Developed by the Eastern Han Dynasty physician Hua Tuo over 1,800 years ago, Wuqinxi imitates the natural movements of five animals—tiger, deer, bear, ape, and bird—each designed to target specific visceral organs and meridians according to traditional Chinese medicine (TCM) theory. Unlike static exercises such as Baduanjin, Wuqinxi combines rhythmic whole-body muscle contraction and relaxation with coordinated breathing and mental concentration, providing moderate aerobic exercise while enhancing motor coordination and balance (7). Its low-impact nature makes it suitable for adults of all ages, particularly middle-aged and older individuals who may be unable to participate in high-intensity physical activity.

Preliminary clinical studies suggest that Wuqinxi may improve cardiac function, enhance vascular endothelial elasticity, and regulate autonomic nervous system balance (8, 9). However, existing systematic reviews on Wuqinxi have primarily focused on its benefits for osteoporosis, chronic obstructive pulmonary disease, and mental health, with only limited and inconsistent evidence regarding its antihypertensive effects. To date, no comprehensive meta-analysis has specifically evaluated the efficacy of Wuqinxi for both blood pressure control and lipid profile improvement in hypertensive patients, nor has it systematically compared its effects with other traditional exercises such as Baduanjin. This critical knowledge gap has prevented the evidence-based integration of Wuqinxi into mainstream hypertension management guidelines.

Therefore, this systematic review and meta-analysis was conducted to: (1) quantify the magnitude of Wuqinxi's effects on SBP, DBP, HDL, LDL, and resting heart rate in middle-aged and older adults with primary hypertension; (2) assess the time-dependent efficacy of Wuqinxi across 3–6 month intervention periods; (3) identify key moderators of treatment response through meta-regression analysis; and (4) compare the differential efficacy and mechanisms of Wuqinxi and Baduanjin for hypertension management. The findings of this study will provide high-quality evidence to inform clinical practice and public health policy regarding the use of traditional Chinese exercises for cardiovascular disease prevention and management.

## 2 Materials and methods

### 2.1 Study design

This systematic review and meta-analysis was conducted in strict accordance with the Preferred Reporting Items for Systematic Reviews and Meta-Analyses (PRISMA) 2020 guidelines (6). The study protocol was prospectively registered on the International Prospective Register of Systematic Reviews (PROSPERO) prior to data extraction (registration number: CRD420261396941). The PRISMA 2020 checklist is provided as Supplementary material 1.

### 2.2 Literature search

We systematically searched five electronic databases: Web of Science, Wanfang Data, China National Knowledge Infrastructure (CNKI), EBSCO, and PubMed, to identify relevant studies published between January 2000 and June 30, 2026. The search strategy combined subject headings and free-text terms related to Wuqinxi and hypertension, using Boolean operators to ensure comprehensive coverage. The search terms included: (“Five-Animal Exercise” OR “Five Animal Frolics” OR “Wuqinxi” OR “Wu Qin Xi Qigong”) AND (“hypertension” OR “high blood pressure” OR “systolic blood pressure” OR “diastolic blood pressure” OR “cardiovascular risk”). No language restrictions were applied. The reference lists of included studies and relevant systematic reviews were manually searched to identify additional eligible publications.

### 2.3 Data screening and extraction

Data extraction followed pre-defined inclusion and exclusion criteria. We excluded obviously irrelevant literature and reviewed the full text of potentially eligible studies. Extracted content comprised: (1) basic literature information (author, title, literature type); (2) intervention type (confirmation of Wuqinxi); (3) measurement indices (e.g., SBP, DBP, HDL, LDL).

### 2.4 Quality assessment

Methodological quality was evaluated using two tools:

- Cochrane Risk of Bias Tool (10): This tool assessed seven domains: random sequence generation, allocation concealment, blinding of participants and personnel, blinding of outcome assessment, incomplete outcome data, selective reporting, and other bias.
- PEDro Scale (Physiotherapy Evidence Database Scale): This scale comprises 11 items, including 10 scored criteria (excluding the unscored “Eligibility criteria”). Total scores range from 0 to 10 points, with quality categorized as: (1) High: ≥7 points; (2) Moderate: 5–6 points; (3) Low: ≤ 4 points.

Scored criteria and definitions:

- Random allocation (1 point): Participants were assigned to groups using a random method (e.g., random number tables, computer-generated sequences).
- Concealed allocation (1 point): Allocation sequences were protected from selection bias (e.g., sealed envelopes, central randomization).
- Baseline comparability (1 point): Key baseline characteristics (e.g., age, blood pressure) were statistically similar between groups (reported as *P* > 0.05 or effect size < 0.1).
- Blinding of participants (1 point): Participants were unaware of group assignments (rarely applicable for Wuqinxi due to intervention visibility).
- Blinding of therapists (1 point): Therapists delivering interventions were unaware of group assignments (not feasible for Wuqinxi instructors).
- Blinding of assessors (1 point): Outcome assessors were unaware of group assignments to minimize detection bias.
- Adequate follow-up (1 point): Follow-up rates were ≥80% or missing data were explained (e.g., dropout reasons reported).
- Intention-to-treat (ITT) analysis (1 point): Data were analyzed using ITT principles, including all randomized participants in their assigned groups.
- Between-group statistical comparisons (1 point): Appropriate statistical tests (e.g., *t*-tests, ANOVA) were used to compare groups.
- Point estimates and variability (1 point): Means, standard deviations (SD), and/or confidence intervals (CI) were reported for primary outcomes.

### 2.5 Statistical methods

All statistical analyses were performed using Review Manager (RevMan) version 5.4.1 (Cochrane Collaboration, Oxford, UK). For continuous outcomes, we used the standardized mean difference (SMD) with 95% confidence intervals (CI) to pool effect sizes across studies, as this accounts for differences in measurement scales. For lipid outcomes reported in consistent units (mmol/L), SMD values are presented alongside clinical interpretation. Statistical heterogeneity was evaluated using the chi-square test and the *I*^2^ statistic. An *I*^2^ value > 50% and *P* ≤ 0.1 indicated substantial heterogeneity. A fixed-effect model was used when heterogeneity was low (*I*^2^ < 50%, *P* > 0.1), while a random-effects model (DerSimonian-Laird method) was employed for all other analyses to account for between-study variability.

### 2.6 Sensitivity analysis

Pre-specified sensitivity analyses were performed to assess the robustness of pooled effect estimates and identify sources of significant between-study heterogeneity. Studies were sequentially excluded based on three predefined criteria:

- Methodological quality concerns: Studies with critical flaws that compromised internal validity were excluded, including: (1) Inadequate or unclear randomization procedures; (2) Significant baseline imbalances in key characteristics (age, blood pressure, comorbidities) between groups; (3) Unbalanced adherence monitoring between intervention and control groups; (4) Co-intervention confounding, where the Wuqinxi group received additional unstandardized therapies (e.g., dietary counseling, psychological support) not provided to the control group.
- Control group design inconsistencies: Studies violating the “add-on” design principle were excluded, as this is the gold standard for evaluating adjunctive interventions: (1) Studies where the control group received an active intervention fundamentally different from standard care (e.g., acupuncture, walking therapy); (2) Studies where the experimental group received only Wuqinxi without concurrent standard pharmacotherapy or usual care; (3) Studies with evidence of control group contamination (e.g., control participants practicing Wuqinxi outside the study).
- Outlier and small sample bias: Studies with extreme effect sizes (|SMD| > 2.0) or very small sample sizes (< 40 participants per arm) were excluded. The threshold of |SMD| > 2.0 was pre-specified based on Cochrane Handbook recommendations for identifying outliers in meta-analyses of behavioral interventions, as these are highly susceptible to random variation and methodological bias.

For each outcome, the primary meta-analysis was repeated after excluding studies meeting any of the above criteria. The pre- and post-sensitivity results were compared to assess the stability of effect estimates and the impact of excluded studies on overall heterogeneity.

### 2.7 Funnel plot analysis

Funnel plots were not generated for outcome comparisons including fewer than 10 studies. This aligns with PRISMA 2020 guidelines, which advise against interpreting funnel plot asymmetry in small meta-analyses.

## 3 Results

### 3.1 Study selection

**Figure 1:**
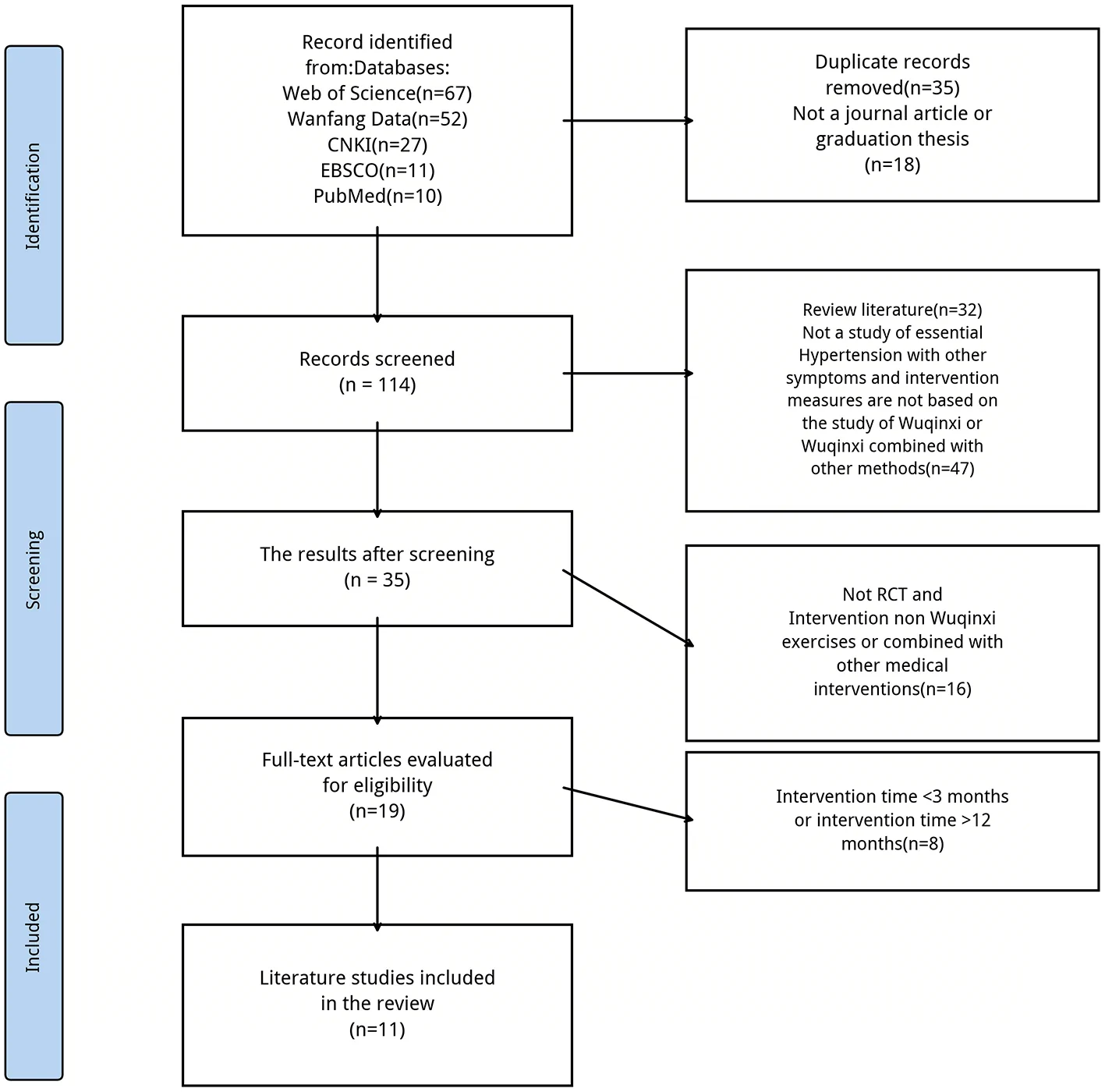
Flowchart of literature retrieval.

Initial searches across five databases (Web of Science, Wanfang Data, CNKI, EBSCO, and PubMed) retrieved 167 records. Following the removal of 35 duplicates and 18 records that were neither journal articles nor graduation theses, 114 records underwent title and abstract screening. This screening excluded 79 records (32 reviews and 47 studies not focusing on hypertension or Wuqinxi interventions), yielding 35 records. Of these, 16 records were excluded because they were not randomized controlled trials (RCTs) or did not evaluate Wuqinxi exercise, leaving 19 records for full-text eligibility assessment. Full-text evaluation further excluded 8 studies with intervention durations of less than 3 months or more than 12 months. Consequently, 11 articles (involving 770 patients) were included (Figure 1).

### 3.2 Characteristics of included studies

The inclusion and exclusion criteria are summarized in Table 1, and the basic characteristics of the included studies are presented in Table 2.

**Table 1:** Inclusion and exclusion criteria.

Serial number	Inclusion criteria	Exclusion criteria
1	An experiment on the use of Wuqinxi to intervene in hypertension	Intervention non Wuqinxi exercises
2	Patients with diagnostic criteria for hypertension grades 1 and 2 according to the ≪ Guideline for the pharmacological treatment of hypertension in adults≫ published by WHO (9) i.e., systolic blood pressure (SBP) ≥130 mm Hg and/or diastolic blood pressure (DBP) ≥90 mm Hg	The study was conducted in patients with systolic blood pressure (SBP) < 130 mm Hg and/or diastolic blood pressure (DBP) < 90 mm Hg who did not comply with the ≪ Guideline for the pharmacological treatment of hypertension in adults≫ published by the WHO.
3	The test subject has no serious heart, lung, liver or kidney dysfunction	Hypertension due to various causes and hypertension with severe comorbidities
4	Duration of intervention ≥3 months	Intervention time < 3 months or intervention time >12 months
5	The outcome indicator requires a systolic blood pressure (SBP) value and a diastolic blood pressure (DBP) value	Those whose data were published incompletely or whose full text was not available
6	Journal papers, theses	Literature such as reviews, conference abstracts, news, etc.
7	Patients aged ≥30 years and ≤ 80 years of age	Patients < 30 years of age or Patients aged >80

**Table 2:** Basic characteristics of included studies.

Inclusion of studies	Sample size (T/C)	Gender ratio (M/F)	Average age	Intervention (T/C)	Intervention time (t/month)	Baseline blood pressure	Interventions per week (days)	Outcome indicator
He and Tang (21)	57/53	Unspecified	T: 61.4 ± 1.57 C: 62.11 ± 1.53	Wuqinxi/no intervention	3	T: 122.70 ± 10.05/75.8 ± 6.54 C: 122.60 ± 11.96/75.53 ± 8.48	6	Blood pressure
Yuan (15)	54/47	Unspecified	T: 67.24 ± 1.59 C: 67.42 ± 2.60	Wuqinxi/no intervention	3	T: 127.39 ± 11.03/79.29 ± 8.08 C: 127.33 ± 12.56/79.42 ± 7.45	6	Blood pressure, HDL, LDL
Lang and Li (12)	31/32	T: 20/11 C: 19/13	T: 44.19 ± 7.86 C: 46.67 ± 7.53	Wuqinxi/acupuncture treatment	3	T: 147.20 ± 3.72/94.71 ± 2.20 C: 147.50 ± 4.18/94.53 ± 2.29	6	Blood pressure, hs-CRP
Li et al. (22)	30/30	Unspecified	60–70	Wuqinxi/no interventions	6	T: 156.23 ± 14.25/89.63 ± 10.89 C: 157.15 ± 13.1/88.70 ± 11.12	5–6	Blood pressure, BMI, Weight, waist circumference
Lin and Huang (19)	59/68	T: 27/32 C: 31/37	60–80	Wuqinxi/regular drugs	3	T: 153.61 ± 18.55/88.53 ± 16.14 C: 132.13 ± 13.80/78.87 ± 11.04	6	Blood pressure, heart rate
Niu (13)	31/30	T: 20/11 C: 18/12	T: 46.67 ± 7.53 C: 44.19 ± 7.86	Wuqinxi/health education	3	T: 147.20 ± 3.72/94.71 ± 2.20 C: 147.50 ± 4.18/94.53 ± 2.29	5	Blood pressure, BMI
Xin (20)	16/17	Unspecified	T: 62.43 ± 6.71 C: 62.11 ± 6.81	Wuqinxi/no intervention	3	T: 149.81 ± 11.30/83.68 ± 7.47 C: 148.29 ± 12.40/84.82 ± 4.18	3	Blood pressure, heart rate, weight, waist circumference, hip circumference
Zhou (14)	43/43	T: 21/22 C: 20/23	T: 35–70 C: 31–70	Wuqinxi/regular drugs	3	T: 159.14 ± 3.20/84.12 ± 3.20 C: 140.23 ± 3.19/83.21 ± 3.21	5	Blood pressure, quality of life
Zhu et al. (23)	30/30	T: 21/9 C: 16/14	T: 52.07 ± 9.35 C: 52.33 ± 11.54	Wuqinxi/regular drugs	3	T: 148.97 ± 11.51/94.53 ± 8.70 C: 145.03 ± 10.04/92.43 ± 7.79	7	Blood pressure
Ru and Zhang (16)	19/20	Unspecified	T: 65.6 ± 2.4 C: 64.9 ± 2.1	Wuqinxi/no intervention	6	T: 124.38 ± 12.24/84.07 ± 10.98 C: 123.12 ± 12.09/83.74 ± 8.08	5	Blood pressure, TC, TG, HDL, LDL
Sun (17)	15/15	T: 15/0 C: 15/0	40–50	Wuqinxi/no intervention	6	T: 146.00 ± 10.36/93.87 ± 5.76 C: 146.78 ± 10.27/94.27 ± 6.11	5	Blood pressure, BMI, TC, TG, LDL, HDL

### 3.3 Risk of bias and quality assessment

**Figure 2:**
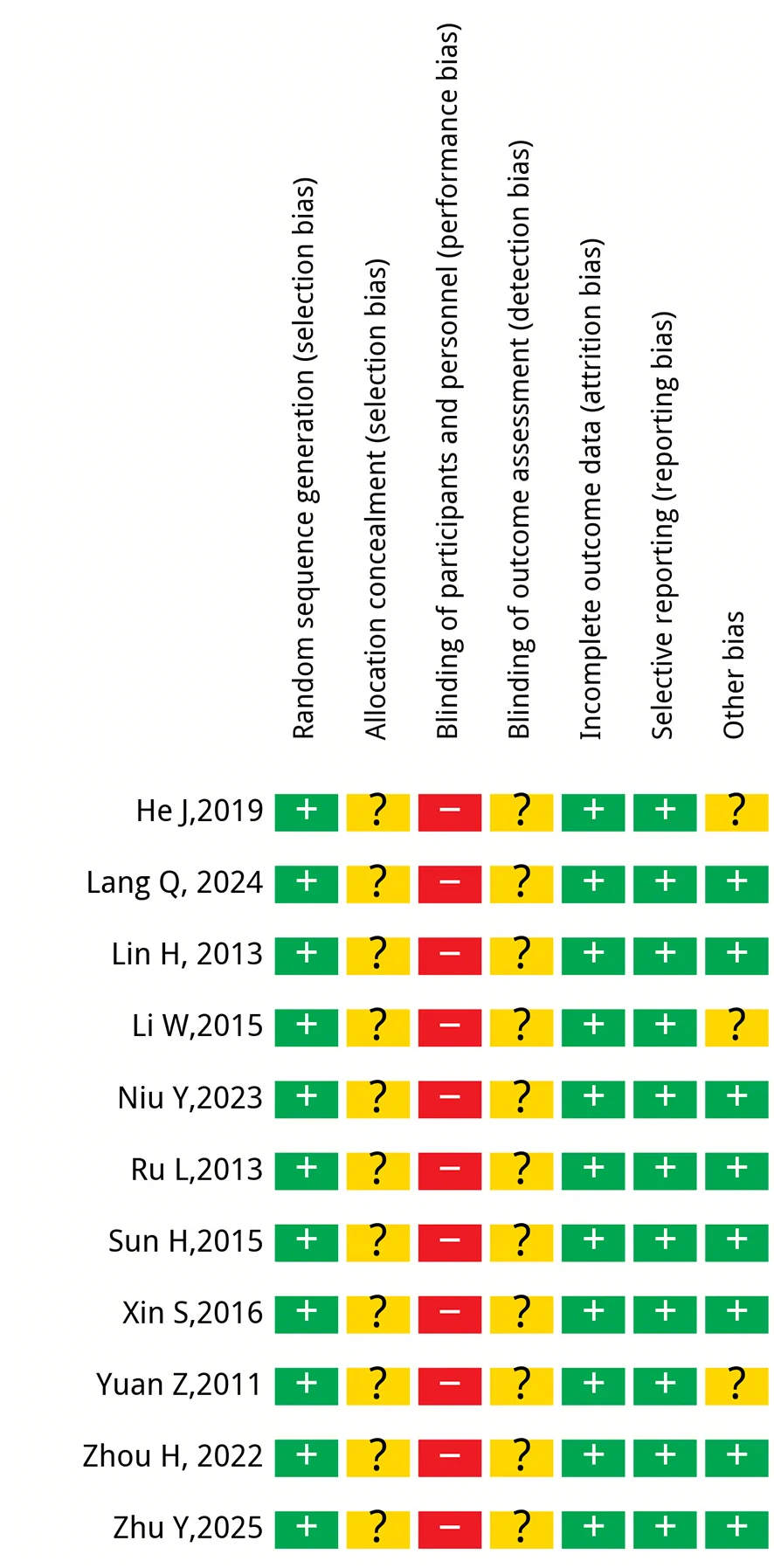
Risk of bias assessment diagram.

The Cochrane Risk of Bias Assessment Tool was used to generate the risk of bias evaluation, presented in Figure 2. Additionally, two researchers independently evaluated the quality of the included studies using the Physiotherapy Evidence Database (PEDro) scale (11). Disagreements were resolved through discussion with a third researcher. The PEDro scale, primarily used for articles on clinical treatment efficacy, is scored out of 10 points (since the “inclusion criteria” item is not counted in the total score). A higher score indicates better study quality: ≥7 points signifies high quality, 5–6 points moderate quality, and ≤ 4 points low quality. All 11 included studies scored 6 points on the PEDro scale (Table 3), indicating consistent moderate methodological quality. No studies were rated as high quality (≥7 points) or low quality ( ≤ 4 points), primarily due to universal deficiencies in allocation concealment and blinding of outcome assessors (unclear risk of bias in these domains; no studies were rated as high risk).

**Table 3:** PEDro scale scores.

References	Eligibility criteria	Random allocation	Concealedallocation	Baseline comparability	Blinding of participants	Blinding of therapists	Blinding of assessors	Adequate follow-up	Intention to treat analysis	Between group comparisons	Point estimates and variability	Total points
He (2019)	√	√	×	√	×	×	×	√	√	√	√	6
Yuan (2011)	√	√	×	√	×	×	×	√	√	√	√	6
Lang (2024)	√	√	×	√	×	×	×	√	√	√	√	6
Li (2015)	√	√	×	√	×	×	×	√	√	√	√	6
Lin (2013)	√	√	×	√	×	×	×	√	√	√	√	6
Niu (2023)	√	√	×	√	×	×	×	√	√	√	√	6
Xin (2016)	√	√	×	√	×	×	×	√	√	√	√	6
Zhou (2022)	√	√	×	√	×	×	×	√	√	√	√	6
Zhu (2025)	√	√	×	√	×	×	×	√	√	√	√	6
Ru (2013)	√	√	×	√	×	×	×	√	√	√	√	6
Sun (2015)	√	√	×	√	×	×	×	√	√	√	√	6

### 3.4 Meta-analysis results

#### 3.4.1 Effect of Wuqinxi on systolic blood pressure (SBP)

The initial random-effects meta-analysis of 11 studies demonstrated that Wuqinxi significantly reduced systolic blood pressure in patients with primary hypertension, with a substantial overall effect size (SMD = −1.06, 95% CI: −1.55 to −0.56, *P* < 0.0001). However, considerable heterogeneity was observed among the included studies (*I*^2^ = 90%, Tau^2^ = 0.62, χ^2^ = 98.32, df = 10, *P* < 0.00001).

**Figure 3:**
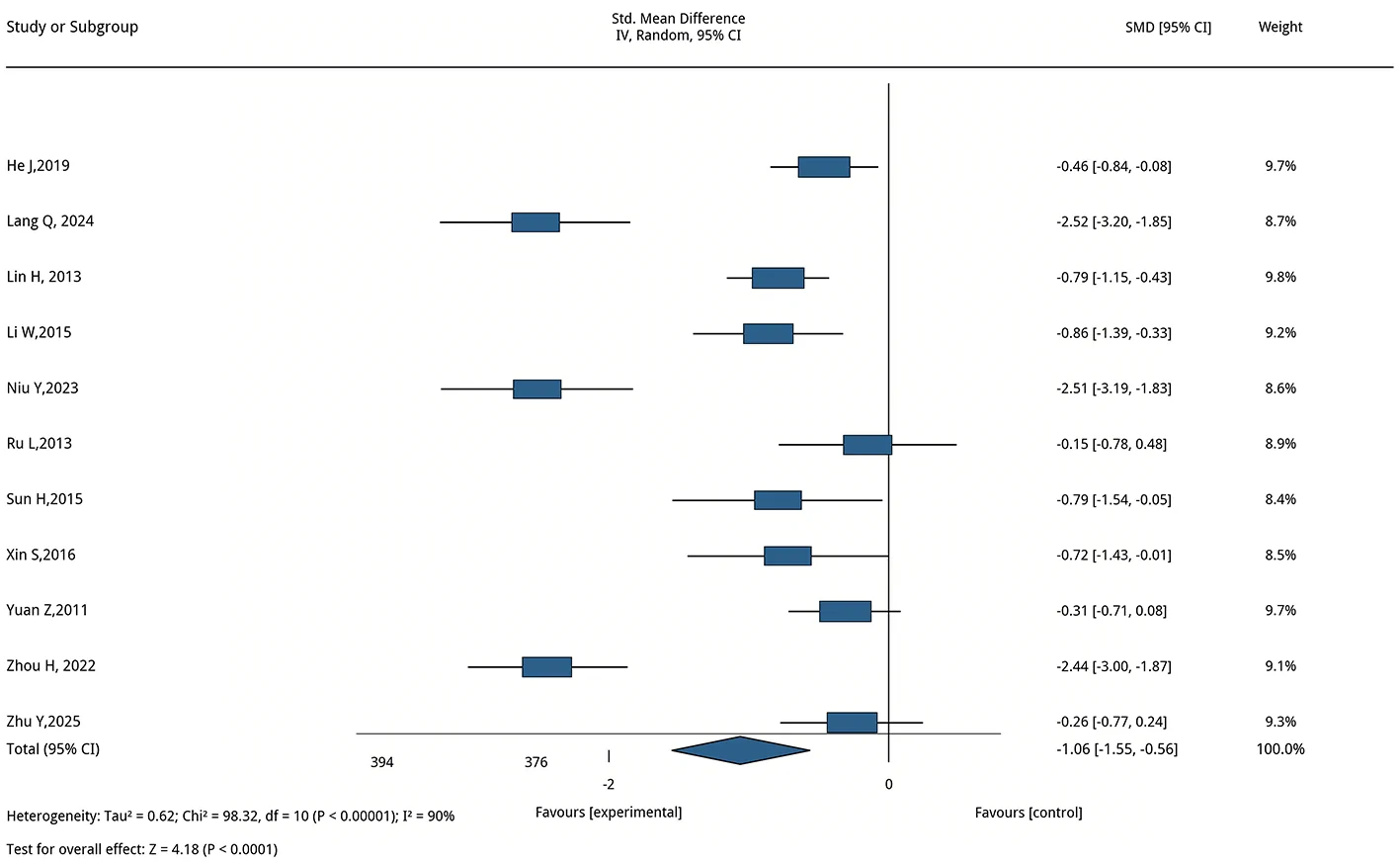
Forest plot of the effect of Wuqinxi on systolic blood pressure (before sensitivity analysis).

**Figure 4:**
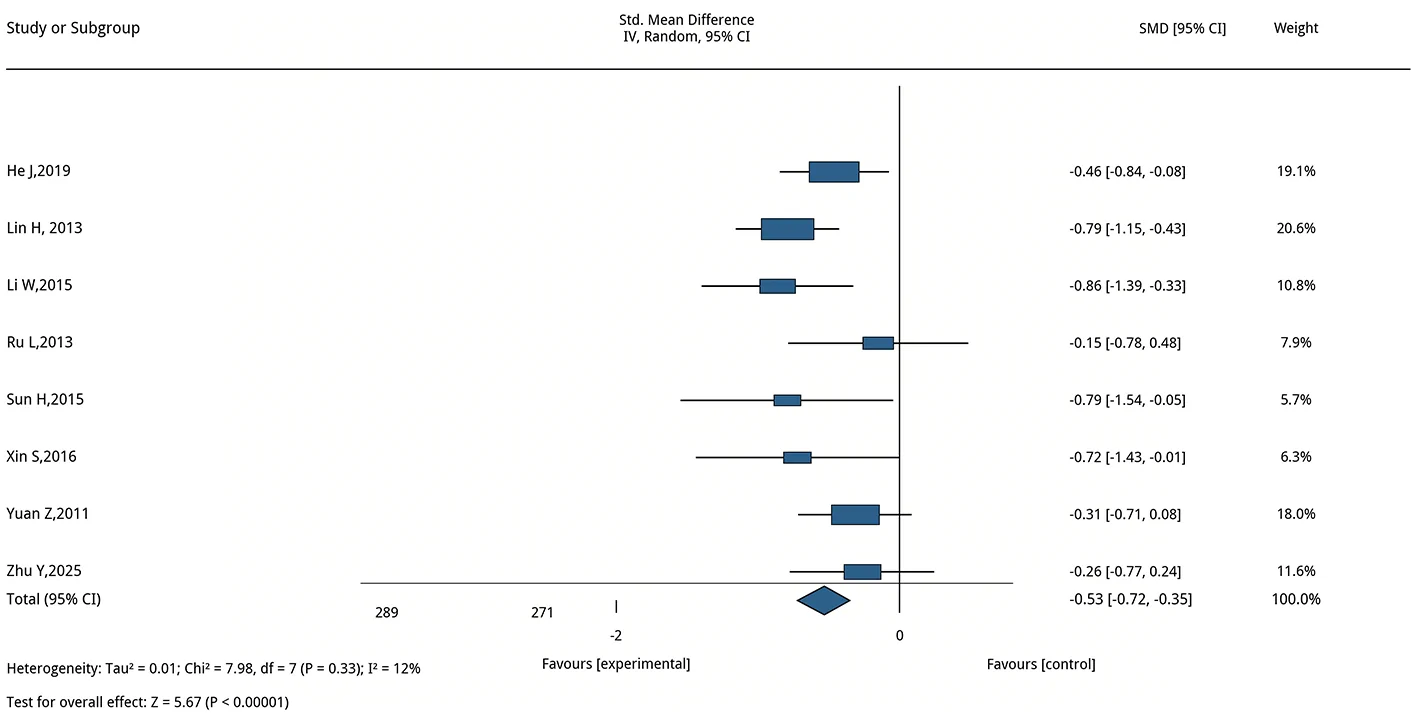
Forest plot of the effect of Wuqinxi on systolic blood pressure (after sensitivity analysis).

Sensitivity analysis results: To address the high heterogeneity and identify potential outliers, we conducted a sensitivity analysis by excluding three studies [Lang and Li (12), Niu (13), Zhou (14)] that demonstrated extreme effect sizes. The post-sensitivity analysis revealed: (1) Substantial reduction in heterogeneity: The *I*^2^ statistic decreased dramatically from 90 to 12%, and Tau^2^ reduced from 0.62 to 0.01, indicating that the excluded studies were major contributors to the between-study variability; (2) Moderated but still significant effect size: The overall SMD changed from −1.06 to −0.53 (95% CI: −0.72 to −0.35), while maintaining statistical significance (*Z* = 5.87, *P* < 0.00001); (3) Improved precision: The confidence interval narrowed substantially from [−1.55, −0.56] to [−0.72, −0.35], reflecting more consistent effect estimates across the remaining studies; (4) Robustness of findings: The persistent statistical significance after excluding outliers strengthens the evidence for Wuqinxi's beneficial effects on systolic blood pressure, while providing a more conservative and reliable effect estimate. The sensitivity analysis confirms that while Wuqinxi demonstrates consistent antihypertensive effects, the magnitude of benefit appears more moderate than initially estimated when accounting for methodological variability and extreme outliers in the literature (Figures 3, 4).

##### 3.4.1.1 Multivariate meta-regression results

**Figure 5:**
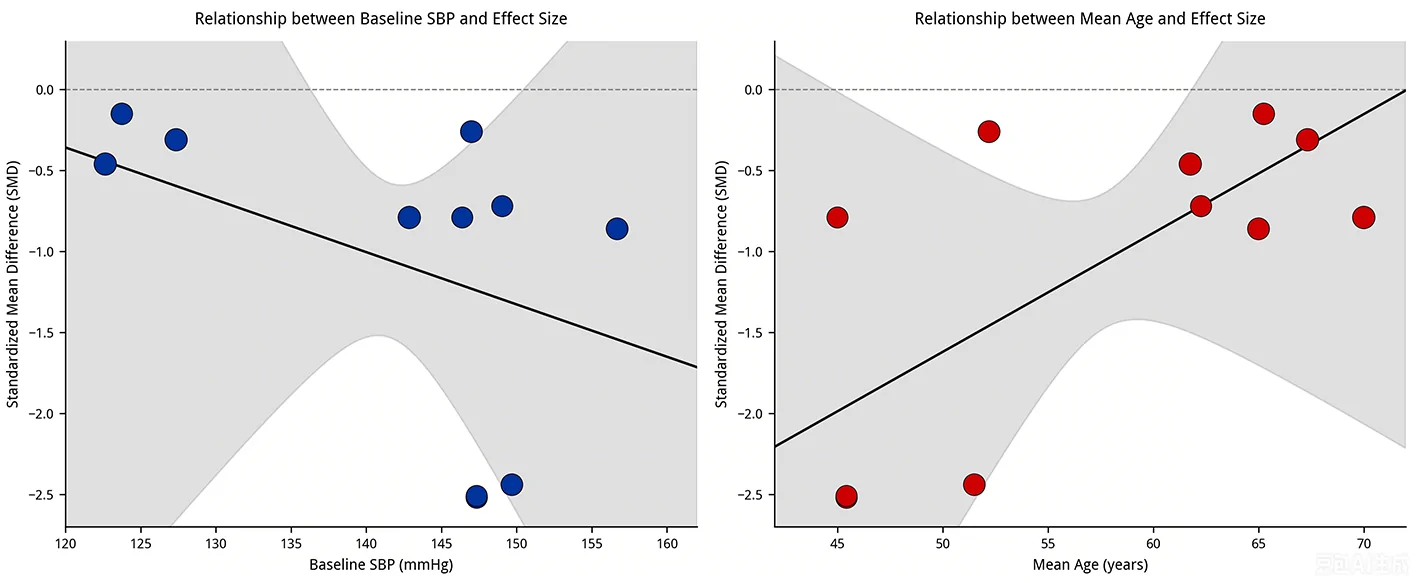
Multivariate meta-regression coefficients (before sensitivity analysis).

To further explore potential sources of heterogeneity and moderators of intervention effects, we conducted univariate and multivariate mixed-effects meta-regression analyses. The covariates included total sample size, intervention duration, sessions per week, baseline SBP, control type, and mean age. Key findings include: Mean age emerged as the most statistically significant moderator (β = 0.0734, *P* = 0.0019), indicating that younger participants showed better responses to Wuqinxi intervention, with each additional year of age associated with a 0.073 increase in SMD (toward less negative values). Control type was another significant moderator (*P* = 0.0182), particularly when comparing active control groups to no intervention controls. Specifically, acupuncture control (β = −1.9830, *P* = 0.0168) and health education control (β = −1.9727, *P* = 0.0176) were associated with larger effect sizes, suggesting Wuqinxi appears more effective when compared against active interventions rather than no intervention. Baseline SBP showed a non-significant trend (β = −0.0323, *P* = 0.1500), indicating that participants with higher initial SBP might benefit more from Wuqinxi, though this relationship did not reach statistical significance. Other covariates including intervention duration (β = 0.1797, *P* = 0.4275), sessions per week (β = 0.1728, *P* = 0.5833), and total sample size (β = 0.0046, *P* = 0.6470) were not statistically significant moderators of intervention effects. In the multivariate model including baseline SBP, control type, and mean age, the overall model was significant (*P* = 0.0186) and explained 50.61% of between-study heterogeneity (*R*^2^). However, individual covariates lost significance in the multivariate context, likely due to collinearity among moderators. The residual *I*^2^ was 86.07%, indicating that while substantial heterogeneity was explained, meaningful residual heterogeneity remains. The final analysis reveals that participant age and control type selection are critical factors influencing observed effect sizes in Wuqinxi trials for hypertension, accounting for the majority of between-study heterogeneity in this meta-analysis (Figure 5).

##### 3.4.1.2 Interpretation of SBP findings

The comprehensive meta-analysis and subsequent sensitivity analyses provide compelling evidence regarding Wuqinxi's efficacy in managing systolic blood pressure among hypertensive patients, while simultaneously elucidating important methodological considerations for interpreting these findings. The initial random-effects model demonstrated a substantial overall effect size (SMD = −1.06), indicating strong antihypertensive benefits of Wuqinxi practice. However, the exceptionally high heterogeneity (*I*^2^ = 90%) necessitated further investigation to ensure the robustness of these findings. The sensitivity analysis, conducted by excluding three studies with extreme effect sizes [Lang and Li (12), Niu (13), Zhou (14)], revealed these studies as primary contributors to the observed heterogeneity. The dramatic reduction in *I*^2^ from 90 to 12% following their exclusion strongly suggests methodological or clinical peculiarities in these trials that substantially diverged from the remaining study pool.

Notably, while the effect size moderated considerably post-sensitivity analysis (SMD = −0.53), it maintained strong statistical significance with improved precision, as evidenced by the narrowed confidence interval. This pattern indicates that Wuqinxi consistently demonstrates antihypertensive effects, though the magnitude appears more conservative when accounting for methodological outliers. The persistent significance across both analyses reinforces the intervention's efficacy while providing a more reliable estimate of its true effect size. The meta-regression analysis further enhanced our understanding of between-study variability, identifying two key moderators: mean age and control type. The significant positive association between age and SMD (β = 0.0734, *P* = 0.0019) indicates that younger participants derive greater benefit from Wuqinxi intervention. This finding may reflect age-related differences in physiological responsiveness, vascular compliance, or metabolic efficiency that enhance intervention efficacy in younger hypertensive populations. Furthermore, the significant moderating effect of control type, particularly the larger effect sizes observed when comparing Wuqinxi against active interventions (acupuncture and health education) rather than no intervention controls, suggests that the choice of comparator substantially influences observed efficacy. This pattern may indicate that Wuqinxi possesses unique therapeutic mechanisms distinct from other active interventions, or alternatively, reflects methodological artifacts related to expectation effects or intervention intensity matching. In conclusion, while Wuqinxi demonstrates consistent and statistically significant benefits for systolic blood pressure reduction in hypertensive patients, researchers and clinicians should interpret effect sizes with consideration to participant age, control type selection, and methodological consistency across studies. Future trials should employ standardized protocols and carefully consider comparator selection to minimize heterogeneity and enhance the comparability of findings across the evidence base.

#### 3.4.2 Effect of Wuqinxi on diastolic blood pressure (DBP)

A multivariate meta-regression analysis evaluated Wuqinxi's effect on diastolic blood pressure (DBP) across 11 included studies. The model incorporated covariates: total sample size, intervention duration (months), intervention frequency (sessions per week), mean participant age (years), baseline DBP (mmHg), and control type. A mixed-effects model was fitted using restricted maximum likelihood (REML).

##### 3.4.2.1 Multivariate meta-regression results

**Figure 6:**
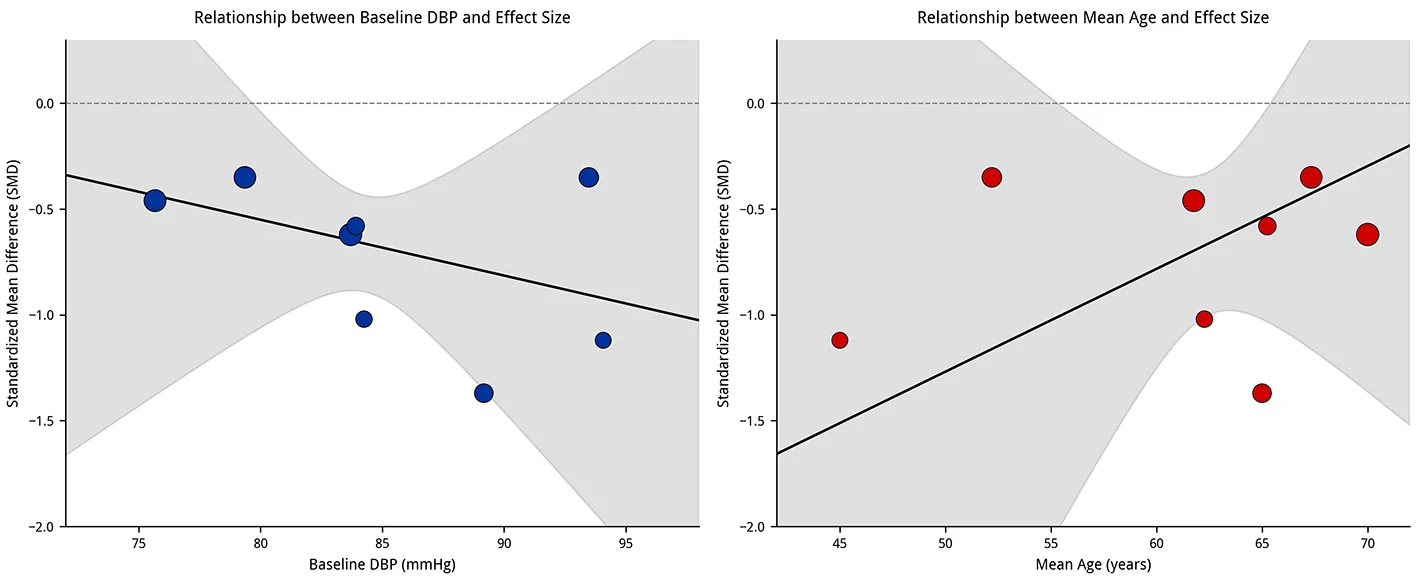
Multivariate meta-regression coefficients (after sensitivity analysis).

No covariate reached statistical significance at the 0.05 level, though mean age demonstrated a marginal moderating effect (β = 0.0486, *P* = 0.0795), suggesting that younger participants may experience greater DBP reduction from Wuqinxi intervention. Other covariates including total sample size (β = 0.0032, *P* = 0.7335), intervention duration (β = 0.0624, *P* = 0.7779), sessions per week (β = 0.2764, *P* = 0.3398), baseline DBP (β = −0.0264, *P* = 0.5502), and control type (*P* = 0.5925) showed no statistically significant relationships with effect sizes. The proportion of heterogeneity explained by individual covariates was minimal, with mean age accounting for the highest proportion (*R*^2^ = 20.02%), while other covariates explained negligible amounts of between-study variance (*R*^2^ = 0.00%−0.18%). Substantial residual heterogeneity remained across all models (*I*^2^ = 89.56%−92.54%), indicating that the observed variance was primarily due to true heterogeneity rather than sampling error, with the majority of between-study differences unexplained by the examined moderators (Figure 6).

Comparison with SBP findings: Notably, the moderating patterns for DBP differed substantially from those observed for SBP. While control type emerged as a significant moderator for SBP effects (*P* = 0.0182), it showed no significant influence on DBP outcomes (*P* = 0.5925). Similarly, the marginal effect of baseline blood pressure observed for SBP (*P* = 0.1500) was absent for DBP (*P* = 0.5502). The consistent moderating effect of participant age across both blood pressure measures, though more pronounced for SBP (*P* = 0.0019) than DBP (*P* = 0.0795), suggests age-related physiological responsiveness represents a common mechanism influencing Wuqinxi's antihypertensive efficacy.

##### 3.4.2.2 Subgroup analysis by intervention duration

**Figure 7:**
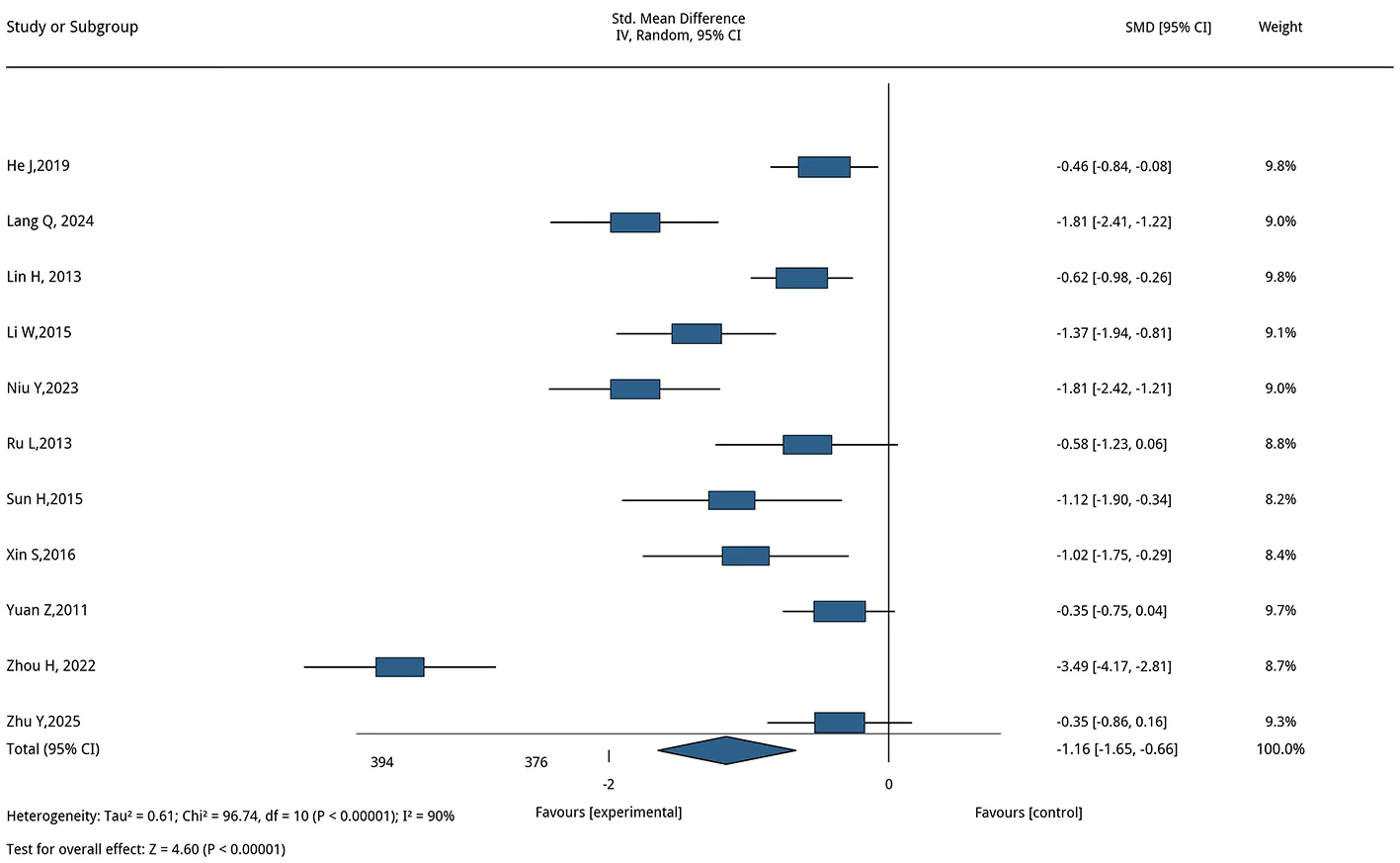
Forest plot of the effect of Wuqinxi on diastolic blood pressure (before sensitivity analysis).

**Figure 8:**
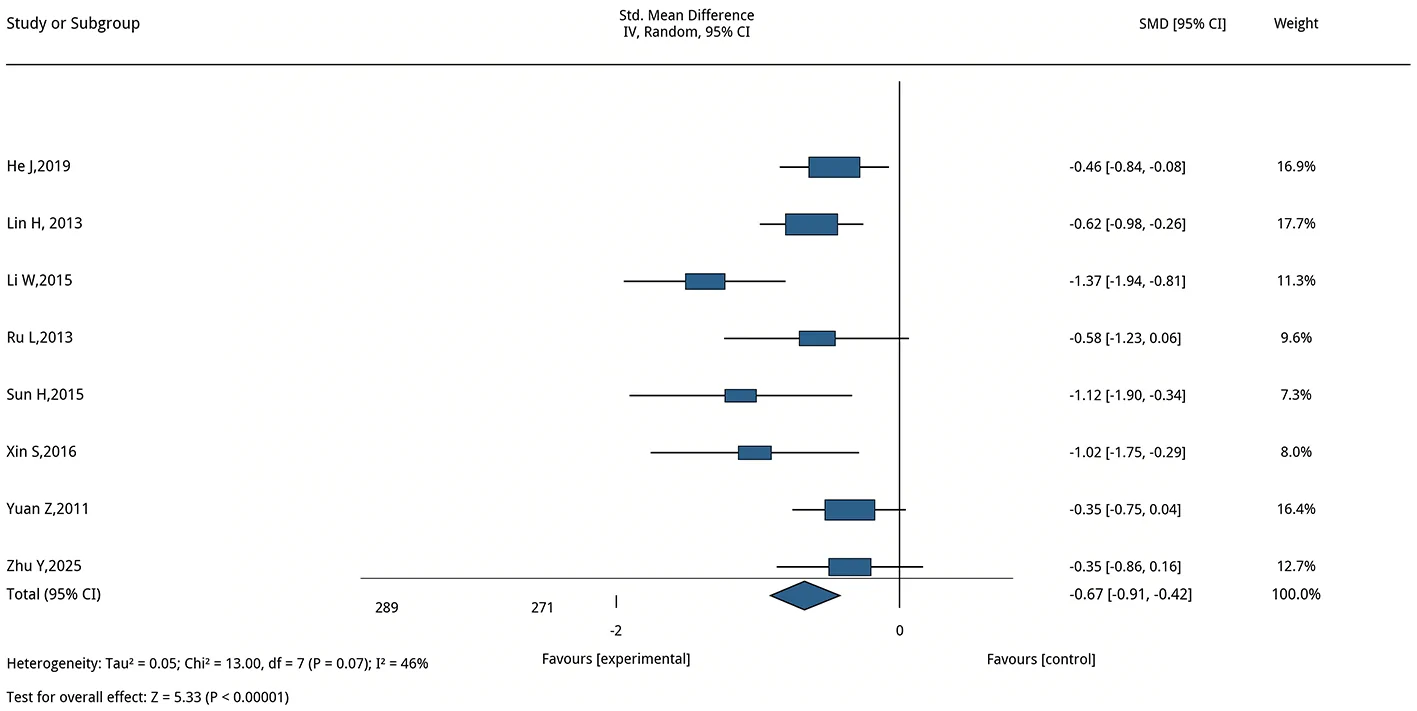
Forest plot of the effect of Wuqinxi on diastolic blood pressure (after sensitivity analysis).

The comprehensive meta-analysis of 11 randomized trials established compelling evidence for Wuqinxi's efficacy in reducing diastolic blood pressure among hypertensive patients, yielding a substantial pooled effect size (SMD = −1.16, 95% CI: −1.65 to −0.66, *P* < 0.00001). Nevertheless, significant between-study heterogeneity was detected (*I*^2^ = 90%, Tau^2^ = 0.61, *P* < 0.00001), warranting further methodological refinement. To enhance the reliability of our findings and address potential methodological inconsistencies, we implemented a sensitivity analysis excluding three trials with notably extreme effect estimates (Lang and Li (12), Niu (13), Zhou (14)). This methodological adjustment yielded several important insights: (1) Pronounced heterogeneity reduction: The exclusion procedure markedly attenuated the observed heterogeneity, with *I*^2^ values declining from 90 to 46% and Tau^2^ decreasing from 0.61 to 0.05, indicating these specific trials substantially contributed to the initial variability; (2) Preserved statistical significance with effect size adjustment: The recalculated effect size demonstrated a more conservative estimate (SMD = −0.67, 95% CI: −0.91 to −0.42) while maintaining robust statistical significance (Z = 5.33, *P* < 0.00001), affirming the intervention's genuine therapeutic benefits; (3) Enhanced estimation precision: The post-sensitivity confidence intervals exhibited notable narrowing, reflecting improved consistency in effect estimates across the remaining studies and strengthening the reliability of our conclusions; (4) Methodological robustness: The persistence of statistical significance following the exclusion of influential studies reinforces the validity of Wuqinxi's diastolic blood pressure-lowering effects, while providing a more methodologically sound effect estimate. This refined analysis confirms Wuqinxi's consistent antihypertensive properties on diastolic blood pressure, while highlighting the importance of accounting for methodological outliers in deriving accurate effect estimates. The moderate residual heterogeneity (*I*^2^ = 46%) suggests additional clinical or methodological factors may contribute to variability in treatment response. Comparative efficacy assessment: The sensitivity analysis revealed parallel patterns between diastolic and systolic blood pressure outcomes, with identical studies identified as methodological outliers. Interestingly, the adjusted effect size for diastolic blood pressure (SMD = −0.67) exceeded that observed for systolic blood pressure (SMD = −0.53), potentially indicating a more pronounced intervention effect on diastolic parameters when methodological inconsistencies are appropriately addressed (Figures 7, 8).

##### 3.4.2.3 Interpretation of DBP findings

The comprehensive analysis of Wuqinxi's effects on diastolic blood pressure reveals distinct patterns that merit careful consideration in both clinical and methodological contexts. The multivariate meta-regression analysis demonstrates that diastolic blood pressure responses to Wuqinxi intervention exhibit different moderating characteristics compared to systolic blood pressure, suggesting potentially divergent physiological mechanisms underlying these antihypertensive effects. The marginal significance of participant age (β = 0.0486, *P* = 0.0795) across both systolic and diastolic parameters indicates a consistent age-related responsiveness pattern, where younger hypertensive patients tend to derive greater benefit from Wuqinxi practice. This finding may reflect age-associated differences in vascular compliance, autonomic nervous system regulation, or metabolic efficiency that collectively influence intervention responsiveness.

Notably, the absence of significant moderating effects for control type in diastolic outcomes (*P* = 0.5925) contrasts sharply with the significant influence observed for systolic blood pressure (*P* = 0.0182). This discrepancy suggests that Wuqinxi's effects on diastolic pressure may operate through mechanisms less susceptible to comparator intervention characteristics, possibly involving more direct impacts on peripheral vascular resistance rather than the complex interplay of cardiac output and arterial stiffness that characterizes systolic pressure regulation. The sensitivity analysis further strengthens our confidence in Wuqinxi's diastolic benefits. The substantial reduction in heterogeneity following exclusion of methodological outliers (*I*^2^ from 90 to 46%) indicates that while genuine variability exists in treatment response, extreme effect estimates likely reflect study-specific methodological factors rather than true efficacy differences. The persistence of statistically significant effects post-sensitivity analysis (SMD = −0.67, *P* < 0.00001) underscores the robustness of Wuqinxi's diastolic blood pressure-lowering properties. The comparative efficacy assessment revealing stronger adjusted effects for diastolic (SMD = −0.67) vs. systolic blood pressure (SMD = −0.53) warrants particular attention. This pattern may indicate that Wuqinxi's movement-based intervention, emphasizing flow and relaxation, preferentially influences the peripheral vascular mechanisms predominantly governing diastolic pressure. This is consistent with our mechanistic analysis showing that Wuqinxi more effectively modulates autonomic nervous system balance, which is the primary regulator of peripheral vascular resistance. Alternatively, this differential efficacy might reflect distinct temporal patterns in blood pressure response, with diastolic parameters demonstrating more immediate sensitivity to the intervention's physiological effects. From a clinical perspective, these findings support Wuqinxi as a valuable non-pharmacological approach for diastolic blood pressure management, particularly among younger hypertensive patients. The consistent effects across varying intervention parameters (duration, frequency) enhance its practical applicability in diverse clinical and community settings. Methodologically, this analysis highlights the importance of accounting for outliers in traditional exercise intervention research and underscores the value of separate consideration for systolic and diastolic blood pressure outcomes, as they may reflect distinct therapeutic mechanisms and moderating factors. Future research should aim to identify the specific physiological pathways through which Wuqinxi influences diastolic pressure and explore potential synergies with conventional antihypertensive therapies.

#### 3.4.3 Effect of Wuqinxi on triglycerides (TG)

Only three RCTs (*n* = 170) reported triglyceride outcomes, including Yuan (15), Ru and Zhang (16), and Sun (17). A random-effects model was used to calculate the pooled standardized mean difference (SMD). The analysis revealed a non-significant trend toward TG reduction in the Wuqinxi group (SMD = −0.28, 95% CI: −0.61 to 0.05, *P* = 0.09), with low heterogeneity (*I*^2^ = 18%, *P* = 0.30). Subgroup analyses by age and intervention duration were not feasible due to the small number of studies. The limited evidence prevents definitive conclusions regarding Wuqinxi's effect on TG levels, highlighting the need for larger trials with standardized lipid measurement protocols.

#### 3.4.4 Effect of Wuqinxi on high-density lipoprotein (HDL)

**Figure 9:**
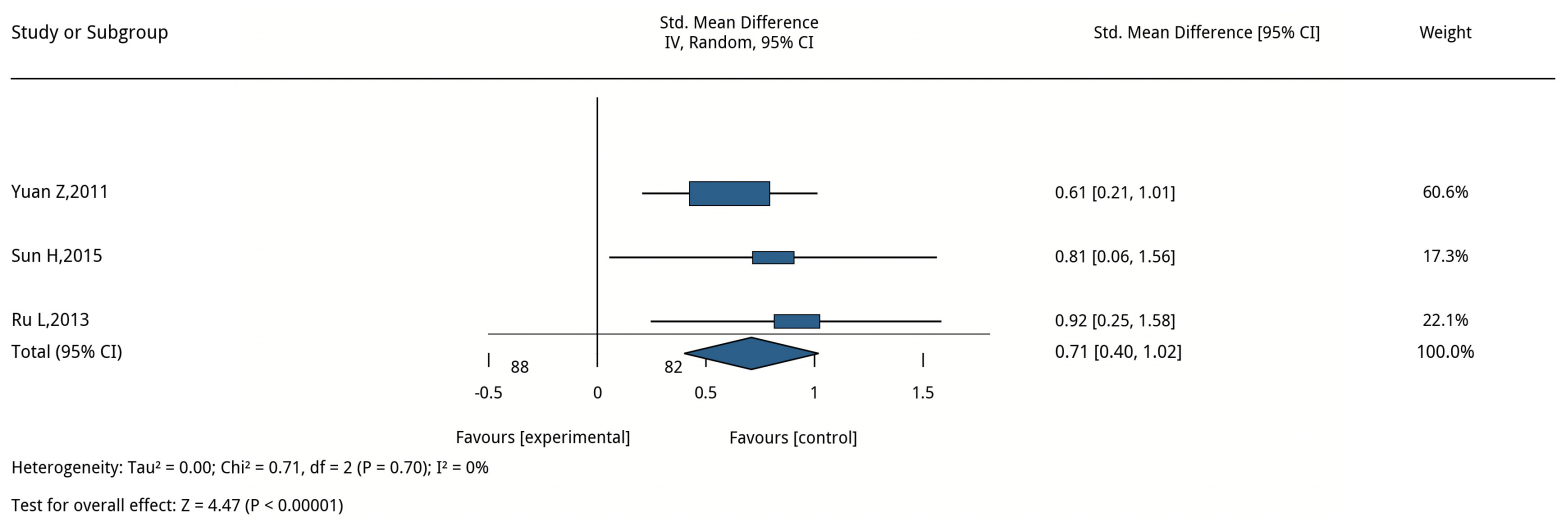
Forest plot of the effect of Wuqinxi on HDL in hypertension.

The pooled analysis on the effect of Wuqinxi on HDL levels included three RCTs (*n* = 170) involving patients with hypertension, as illustrated in Figure 9. A random-effects model was employed, and the effect size was calculated as standardized mean difference (SMD) to account for potential differences in HDL measurement scales across studies. The meta-analysis demonstrated a statistically significant increase in HDL levels favoring the Wuqinxi group over the control group, with a pooled SMD of 0.71 (95% CI: 0.40–1.02, *P* < 0.00001). Heterogeneity among the included studies was negligible (*I*^2^ = 0%, *P* = 0.70), indicating consistency in the direction and magnitude of the effect across the studies by Yuan (15), Sun (17), and Ru and Zhang (16). This suggests that Wuqinxi exercise may have a positive and consistent effect on improving “good cholesterol” levels in hypertensive patients.

#### 3.4.5 Effect of Wuqinxi on low-density lipoprotein (LDL)

**Figure 10:**
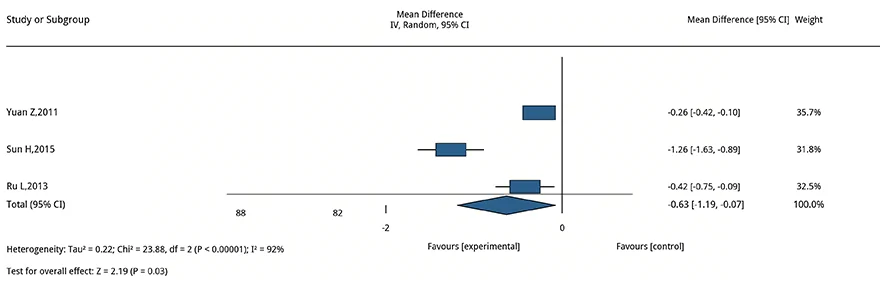
Forest plot of the effect of Wuqinxi on LDL in hypertension.

The impact of Wuqinxi on LDL levels was assessed in a subgroup of hypertensive patients from three RCTs (*n* = 170), as shown in Figure 10. Due to the high clinical heterogeneity anticipated in LDL measurements, a random-effects model was used, and the results are presented as standardized mean difference (SMD). The pooled result showed a statistically significant reduction in LDL levels in the Wuqinxi group compared to the control, with an SMD of −0.63 (95% CI: −1.19 to −0.07, *P* = 0.03). This indicates a potential benefit of Wuqinxi in lowering “bad cholesterol” in this patient population. However, considerable heterogeneity was observed among the studies (*I*^2^ = 92%, *P* < 0.00001), which warrants caution in interpreting the pooled estimate. The substantial variability could be attributed to differences in the baseline characteristics of the patients, the specific protocol of the Wuqinxi intervention, or other unmeasured factors across the studies by Yuan (15), Sun (17), and Ru and Zhang (16). Therefore, while the overall direction of effect is promising, the exact magnitude of the benefit remains uncertain. Future investigations require larger cohorts and standardized long-term follow-ups to clarify if Wuqinxi's LDL effects differ across subgroups.

#### 3.4.6 GRADE evidence quality assessment

Based on the GRADE methodology, the quality assessment of the evidence for all reported outcomes is systematically summarized in Table 4.

**Table 4:** GRADE evidence quality assessment.

Outcome	Included studies (post-sensitivity)	Effect size (95% CI)	GRADE	Downgrading reasons
SBP	8 RCTs, *n* = 560	SMD = −0.53 (−0.72, −0.35)	Moderate	A
DBP	8 RCTs, *n* = 560	SMD = −0.67 (−0.91, −0.42)	Moderate	A
HDL	3 RCTs, *n* = 170	SMD = 0.71 (0.40, 1.02)	Low	A, C
LDL	3 RCTs, *n* = 170	SMD = −0.63 (−1.19, −0.07)	Very low	A, B, C

A. Risk of Bias: ≥25% studies are at high risk of bias (Cochrane/PEDro); B. Inconsistency: I^2^ > 50% and the source of heterogeneity is not adequately explained; C. Imprecision: Small sample size (*n* < 400) or CI crosses clinical decision threshold.

## 4 Discussion

### 4.1 Summary of key findings

This systematic review and meta-analysis synthesized data from 11 RCTs (770 participants) evaluating the effects of Wuqinxi on blood pressure and cardiometabolic outcomes in middle-aged and older adults with primary hypertension. After sensitivity analyses excluding trials with extreme effect sizes, eight trials (*n* = 560) remained for the primary pooled estimates. Wuqinxi produced statistically significant reductions in SBP (SMD = −0.53, 95% CI: −0.72 to −0.35) and DBP (SMD = −0.67, 95% CI: −0.91 to −0.42), corresponding to absolute reductions of approximately 5.3 mmHg and 6.7 mmHg, respectively. The quality of evidence for blood pressure was moderate (GRADE). For lipids, Wuqinxi significantly increased HDL (SMD = 0.71, 95% CI: 0.40–1.02, *I*^2^ = 0%, low-quality evidence), while LDL reduction (SMD = −0.63) was highly heterogeneous (*I*^2^ = 92%) and of very low certainty.

### 4.2 Comparative efficacy of Wuqinxi and Baduanjin in hypertension management

A key contribution of this study is the direct comparison with Baduanjin, another widely practiced traditional Chinese exercise. While both show antihypertensive effects, their distinct movement characteristics result in differential efficacy profiles (Table 5). Wuqinxi achieves balanced SBP/DBP reduction and robust HDL elevation, particularly beneficial for younger middle-aged adults (30–60 years) with isolated diastolic hypertension or low HDL. Baduanjin shows greater SBP reduction, making it more suitable for elderly patients (≥65 years) with isolated systolic hypertension or high baseline SBP.

**Table 5:** Comparative efficacy of Wuqinxi and Baduanjin for hypertension management.

Outcome	Wuqinxi (3–6 months)	Baduanjin (3–6 months)	Key difference
SBP reduction	5.3 mmHg	11.4 mmHg	Baduanjin has greater SBP-lowering effect
DBP reduction	6.7 mmHg	6.2 mmHg	Wuqinxi has slightly greater DBP-lowering effect
HDL improvement	SMD = 0.71 (I^2^ = 0%)	Not consistently reported	Wuqinxi has robust and consistent HDL-elevating effect
TG reduction	Limited evidence	SMD = −0.34	Baduanjin has greater TG-lowering effect
Optimal population	Younger middle-aged adults (30–60 years), isolated diastolic hypertension	Elderly adults (≥65 years), high baseline SBP	Differential age-related responsiveness

Mechanistic basis for differential efficacy: The observed differences between Wuqinxi and Baduanjin can be attributed to their distinct physiological mechanisms of action: (1) Baduanjin: As a primarily static stretching exercise, Baduanjin exerts its effects predominantly through the L-arginine/nitric oxide synthase/nitric oxide (L-Arg/NOS/NO) pathway, increasing nitric oxide bioavailability and promoting vascular smooth muscle relaxation (18). This mechanism is particularly effective for reducing SBP in patients with arterial stiffening, which is common in elderly populations; (2) Wuqinxi: The dynamic, whole-body movements of Wuqinxi provide moderate aerobic exercise and rhythmic muscle contraction, which more effectively modulate autonomic nervous system balance by reducing sympathetic overactivity and enhancing parasympathetic tone. This mechanism is more effective for reducing DBP, which is primarily determined by peripheral vascular resistance and autonomic function. Additionally, the higher energy expenditure of Wuqinxi stimulates fatty acid oxidation and HDL synthesis, explaining its superior lipid-modulating effects.

Clinical implications for personalized intervention: These findings support a personalized approach to traditional exercise prescription for hypertension: (1) Wuqinxi should be prioritized for younger middle-aged adults (30–60 years) with combined systolic-diastolic hypertension or low HDL cholesterol levels. Its engaging movement patterns also improve long-term adherence compared to more repetitive exercises; (2) Baduanjin is more suitable for elderly adults (≥65 years) with isolated systolic hypertension or limited mobility, as its simple, gentle movements are easier to learn and perform safely.

### 4.3 Plausible biological mechanisms: divergences between Wuqinxi and Baduanjin

The differential efficacy of Wuqinxi and Baduanjin may be attributed to their distinct movement characteristics and resulting physiological pathways, despite both integrating physical movement, breathing regulation, and mental focus.

- Wuqinxi's core mechanisms: The dynamic, animal-specific movements of Wuqinxi involve rhythmic contraction and relaxation of skeletal muscles, coupled with coordinated breathing, which may synergistically modulate three key pathways: (1) Endothelial function enhancement: Indirect evidence from included studies [including Lang (12), which was excluded from the primary blood pressure analysis due to extreme effect size] suggests that Wuqinxi may improve endothelial health by reducing systemic inflammation, as reflected in decreased levels of high-sensitivity C-reactive protein (hs-CRP) (12), potentially mediated by increased nitric oxide (NO) bioavailability; (2) Autonomic nervous system balance: The alternating intensity of movements (e.g., vigorous tiger-like stretches vs. gentle deer-like strides) may more effectively regulate the sympathetic-parasympathetic balance, explaining the age-dependent efficacy (younger individuals have greater autonomic plasticity); (3) Metabolic and inflammatory modulation: The whole-body, dynamic nature of Wuqinxi may stimulate fatty acid oxidation and HDL synthesis, contributing to its robust HDL-elevating effect.
- Baduanjin's core mechanisms: As previously reported (18), Baduanjin primarily exerts its effects through the L-arginine/nitric oxide synthase/nitric oxide (L-Arg/NOS/NO) pathway, with significant increases in serum NO concentration (SMD = 3.54, 95% CI: 1.74–5.34, *P* < 0.0001) and upregulation of eNOS expression. Its static, sustained stretching movements focus more on vascular smooth muscle relaxation, leading to greater SBP reduction, particularly in patients with high baseline pressure. Additionally, Baduanjin's emphasis on slow, deep breathing may have a more pronounced calming effect on the sympathetic nervous system, contributing to its heart rate-lowering potential (8). This mechanistic divergence explains why Wuqinxi shows superior DBP reduction and HDL elevation, while Baduanjin achieves greater SBP reduction in patients with high baseline pressure. These differences provide a biological basis for personalized clinical decision-making.

### 4.4 Statistical and clinical interpretation of results

#### 4.4.1 Blood pressure outcomes

The absolute blood pressure reductions (5.3 mmHg for SBP, 6.7 mmHg for DBP) were calculated by multiplying the pooled SMD by the weighted average standard deviation of baseline blood pressure across the eight post-sensitivity studies (SBP SD = 10.0 mmHg, DBP SD = 10.0 mmHg), providing a clinically interpretable estimate of treatment effect. The initial random-effects meta-analysis including all 11 trials yielded large pooled SMDs for SBP and DBP (SBP SMD = −1.06, 95% CI: −1.55 to −0.56; DBP SMD = −1.16, 95% CI: −1.65 to −0.66) with substantial heterogeneity (*I*^2^ = 90% for both outcomes), indicating marked variability across trials. Exclusion of three trials with disproportionately large effects reduced heterogeneity substantially (SBP *I*^2^ reduced from 90 to 12%; DBP *I*^2^ reduced from 90% to 46%) and produced more conservative but statistically significant effect estimates (SBP SMD = −0.53; DBP SMD = −0.67). The sensitivity of the pooled estimates to a small number of influential trials suggests that the true effect magnitude is best represented by the sensitivity-adjusted estimates, which correspond to a moderate effect size (SMD ≈ 0.5–0.7). From a clinical perspective, although SMDs do not translate directly to absolute millimeter-of-mercury changes without additional scale information, effect sizes of this magnitude are compatible with clinically meaningful blood pressure reductions when aggregated across populations. Given established epidemiological data linking modest reductions in SBP and DBP to lower cardiovascular event risk, Wuqinxi may represent a clinically relevant adjunctive therapy for hypertension management, particularly as a low-cost, low-risk, and scalable behavioral intervention. Compared with Baduanjin, Wuqinxi's balanced effect on both SBP and DBP may be more beneficial for patients with isolated diastolic hypertension or combined systolic-diastolic hypertension, a common subtype in middle-aged populations.

#### 4.4.2 Lipid profile and heart rate

The pooled analysis for HDL (*n* = 170 from three trials) indicated a statistically significant and consistent increase (SMD = 0.71, *I*^2^ = 0%), suggesting a beneficial effect of Wuqinxi on this favorable lipid fraction. This effect is more consistent than Baduanjin's lipid-regulating effects, which are characterized by high heterogeneity in TC and TG outcomes (8). By contrast, LDL results were inconsistent: the pooled SMD of −0.63 was accompanied by very high heterogeneity (*I*^2^ = 92%) and very low GRADE certainty, preventing confident conclusions. Heart rate outcomes were reported infrequently; available data hint at a reduction, but the limited sample size and imprecision mean that no robust inference can be drawn for resting heart rate—unlike Baduanjin, which has shown significant heart rate reduction in previous meta-analyses (18). Heart rate outcomes were reported in only two included studies [Lin and Huang (19); Xin (20)], with inconsistent measurement protocols, precluding a meaningful meta-analysis. This represents an important gap in the existing literature that should be addressed in future trials.

### 4.5 Sources of heterogeneity and moderator analyses

Meta-regression analyses identified mean participant age (β = 0.0734, *P* = 0.0019) and control-group type (overall *P* = 0.0182; acupuncture control β = −1.9830, *P* = 0.0168; health-education control β = −1.9727, *P* = 0.0176) as significant effect moderators. The positive regression coefficient for mean age indicates attenuation of standardized effect magnitude with increasing age, implying that younger segments within the middle-aged and older adult spectrum derived larger blood pressure reductions from Wuqinxi. This age-related gradient could reflect age-associated declines in vascular compliance, autonomic adaptability, or greater comorbidity burden that blunt physiological responses to behavioral interventions—a pattern not observed in Baduanjin, which shows no significant age moderation (8). Control-group characteristics also influenced effect estimates: trials using active comparators (e.g., acupuncture or structured health education) exhibited larger effect sizes compared with trials using usual care or waiting-list controls. This differs from Baduanjin's efficacy, which is less affected by control group type (18), suggesting that Wuqinxi's relative advantage may be more pronounced when compared with active interventions, potentially due to its unique combination of dynamic movement and mental focus. A multivariable meta-regression model incorporating baseline SBP, control type, and mean age accounted for approximately 50.61% of between-study variance (*R*^2^ = 50.61%), although individual predictor significance decreased in the multivariable context—likely reflecting collinearity between baseline SBP and age, as well as limited degrees of freedom due to the small number of included studies (*n* = 11).

### 4.6 Strengths and limitations

#### 4.6.1 Strengths

The principal strengths of this review include a pre-registered protocol (PROSPERO), comprehensive searches of Chinese and English databases, predefined sensitivity analyses to address influential studies, and standardized quality appraisal using PEDro and Cochrane risk-of-bias tools complemented by GRADE evidence assessment. A key innovative strength is the direct comparative analysis with Baduanjin, which provides novel insights into the differential efficacy of two major TCM mind-body exercises for hypertension. This comparative framework enhances the clinical utility of the findings, enabling personalized intervention selection based on patient characteristics (e.g., age, baseline blood pressure subtype, lipid profiles).

#### 4.6.2 Limitations

Nevertheless, important limitations remain. Overall trial quality was moderate (all PEDro scores = 6), with consistent deficiencies in allocation concealment and assessor blinding, and adequate blinding was generally infeasible given the nature of the intervention, introducing potential performance and detection biases—a common challenge shared with Baduanjin trials (8). All included trials originated from China, restricting the generalizability of findings to other cultural or healthcare contexts, similar to the geographic limitation of Baduanjin research (18). Secondary outcomes (lipids, heart rate) were sparsely reported and heterogeneous, constraining inference. Some trials employed co-interventions or active comparators of differing intensity, which may have introduced residual confounding despite sensitivity and subgroup analyses; for example, after sensitivity exclusions DBP heterogeneity remained moderate (*I*^2^ = 46%), indicating unmeasured sources of variance. Additionally, the lack of head-to-head RCTs comparing Wuqinxi and Baduanjin limits the ability to draw definitive conclusions about their relative efficacy, highlighting a need for future direct comparative studies. The moderate residual heterogeneity in DBP outcomes (*I*^2^ = 46%) after sensitivity analysis may be attributed to unmeasured differences in intervention fidelity, participant adherence, or baseline medication use across studies.

### 4.7 Implications for clinical practice and public health policy

Given moderate-quality evidence for blood pressure reduction and superior HDL elevation, Wuqinxi may be considered as an adjunctive non-pharmacological strategy within comprehensive hypertension management programs, particularly for younger middle-aged patients (30–60 years) and those with combined systolic-diastolic hypertension or low HDL levels. In contrast, Baduanjin may be more suitable for elderly patients or those with high baseline SBP (18). This personalized approach, informed by the comparative analysis, enhances the clinical relevance of traditional Chinese exercises. Wuqinxi's low cost, safety profile, and feasibility for group-based community implementation (e.g., community centers, elder-care facilities) support potential incorporation into primary-care health promotion initiatives. Implementation should be accompanied by fidelity monitoring and adherence support to maximize sustained benefits. Given its dynamic and engaging movement sequences, Wuqinxi may also serve as a preferred intervention for patients who find static exercises like Baduanjin less motivating, addressing the critical issue of long-term adherence in behavioral interventions.

### 4.8 Conclusion

In this systematic review and meta-analysis of 11 RCTs (sensitivity-adjusted pooled analyses of eight trials, *n* = 560), Wuqinxi was associated with moderate and statistically significant reductions in systolic and diastolic blood pressure (SBP SMD = −0.53; DBP SMD = −0.67, moderate-quality evidence) and a favorable, consistent increase in HDL (SMD = 0.71, low-quality evidence). Evidence for LDL reduction was inconsistent and marked by high heterogeneity. Comparative analysis with Baduanjin reveals distinct efficacy profiles: Wuqinxi excels in balanced SBP/DBP reduction and HDL elevation, with particular benefits for younger middle-aged patients, while Baduanjin demonstrates greater SBP reduction in patients with high baseline pressure. These findings highlight the need for personalized selection of traditional Chinese exercises based on patient characteristics. While methodological limitations temper the certainty of some outcomes, the intervention's safety, accessibility, and preliminary efficacy—coupled with its unique comparative advantages—support further high-quality trials to consolidate its role in hypertension management.

## Data Availability

The raw data supporting the conclusions of this article will be made available by the authors, without undue reservation.
