# Supplementary material for: Effects of Wuqinxi on blood pressure and lipids in hypertensive middle-aged and elderly adults: a meta-analysis
Source: Front Med (Lausanne). 2026 Sep 1;13:1929567. doi: 10.3389/fmed.2026.1929567 (PMC13574633; doi:10.3389/fmed.2026.1929567)
Supplement: Supplementary file 1 [file Data_Sheet_1.docx]

# Supplementary File S1. Full Search Strategies Used in This Review

This review conducted comprehensive literature searches across both English and Chinese databases. The search was last updated on June 17, 2026.

## English Databases

### 1. PubMed

Search Strategy:

((Tai ji) OR (Tai ji quan) OR (Tai Chi) OR (Tai ji quan) OR (Tai chi Qigong) OR (Tai chi quan) OR (Tai Chi Chuan) OR (Tai ji Chuan))combined with ((essential hypertension) OR (hypertension) OR (high blood pressure) OR (systolic blood pressure) OR (diastolic blood pressure) OR (blood pressure control) OR (cardiovascular health))

Filters applied: None

Date of last search: June 17, 2026

### 2. Web of Science Core Collection

Search Strategy (Topic field):

((Tai ji) OR (Tai ji quan) OR (Tai Chi) OR (Tai ji quan) OR (Tai chi Qigong) OR (Tai chi quan) OR (Tai Chi Chuan) OR (Tai ji Chuan))combined with ((essential hypertension) OR (hypertension) OR (high blood pressure) OR (systolic blood pressure) OR (diastolic blood pressure) OR (blood pressure control) OR (cardiovascular health))

Indexes searched: SCI-EXPANDED, SSCI, A&HCI, CPCI-S

Date of last search: June 17, 2025

### 3. Scopus

Search Strategy:

((Tai ji) OR (Tai ji quan) OR (Tai Chi) OR (Tai ji quan) OR (Tai chi Qigong) OR (Tai chi quan) OR (Tai Chi Chuan) OR (Tai ji Chuan))combined with ((essential hypertension) OR (hypertension) OR (high blood pressure) OR (systolic blood pressure) OR (diastolic blood pressure) OR (blood pressure control) OR (cardiovascular health))

Document type: Article and Review

Language: No restriction

Date of last search: June 17, 2025

### 4. EBSCOhost (MEDLINE, CINAHL)

Search Strategy:

TX((Five-Animal Exercise) OR (Five Animal Exercises) OR (Five-Animal Qigong) OR (Five Animal Qigong) OR (Five-Animals Frolics) OR (Wu Qin Xi) OR (Wu Qin Xi Qigong) OR (Wuqinxi)) AND (( hypertension) OR (hypertension) OR (high blood pressure) OR (systolic blood pressure) OR (diastolic blood pressure) OR (blood pressure control) OR (cardiovascular health))

Database subset used: MEDLINE Complete, CINAHL Plus

Date of last search: November 8, 2025

## Chinese Databases

### 5. CNKI (中国知网)

检索式:

五禽戏 AND 高血压

资源类型: 学术期刊

语言限制: 中文

检索时间: 2025年11月8日

### 6. Wanfang Data（万方数据）

检索式:

五禽戏 AND 高血压

数据库范围: 医学科技期刊库

检索时间: 2025年11月8日

## Notes

• No language or date restrictions were applied in the initial search.
• Manual searching of reference lists of included articles was also conducted to identify additional relevant studies.
• Duplicate records were removed using EndNote 20.
