# Supplementary material for: Effects of Wuqinxi on blood pressure and lipids in hypertensive middle-aged and elderly adults: a meta-analysis
Source: Front Med (Lausanne). 2026 Sep 1;13:1929567. doi: 10.3389/fmed.2026.1929567 (PMC13574633; doi:10.3389/fmed.2026.1929567)
Supplement: Supplementary file 2 [file Data_Sheet_2.docx]

From: Page MJ, McKenzie JE, Bossuyt PM, Boutron I, Hoffmann TC, Mulrow CD, et al. The PRISMA 2020 statement: an updated guideline for reporting systematic reviews. BMJ 2021;372:n71. doi: 10.1136/bmj.n71. This work is licensed under CC BY 4.0. To view a copy of this license, visit https://creativecommons.org/licenses/by/4.0/

**PRISMA 2020 Checklist**

*Effects of Wuqinxi on blood pressure and lipids in hypertensive middle-aged and elderly: a meta-analysis*

| **Section and Topic** | **Item #** | **Checklist item** | **Location where item is reported** |
| --- | --- | --- | --- |
| **TITLE** |  |  |  |
| Title | 1 | Identify the report as a systematic review. | Title page: "Effects of Wuqinxi on blood pressure and lipids in hypertensive middle-aged and elderly: a meta-analysis" |
| **ABSTRACT** |  |  |  |
| Abstract | 2 | See the PRISMA 2020 for Abstracts checklist. | Abstract: Structured abstract includes Purpose (to quantify effects of Wuqinxi on BP, lipids, and resting heart rate; GRADE evidence certainty), Methods (databases searched: CNKI, PubMed, Web of Science, Embase; January 2000–June 30, 2026; 11 RCTs, 770 participants), Results (numeric SBP/DBP/HDL/LDL findings with GRADE ratings), and Conclusion (Wuqinxi is effective; higher-quality RCTs needed). |
| **INTRODUCTION** |  |  |  |
| Rationale | 3 | Describe the rationale for the review in the context of existing knowledge. | Introduction, first two paragraphs: presents the global burden of hypertension (>1.4 billion adults), limitations of pharmacotherapy (non-adherence, adverse effects), preliminary evidence for Wuqinxi, and the rationale for a comprehensive meta-analysis. |
| Objectives | 4 | Provide an explicit statement of the objective(s) or question(s) the review addresses. | Introduction, final paragraph: explicitly states three objectives—(1) quantify the magnitude of Wuqinxi's effects on SBP, DBP, HDL, LDL, and resting heart rate; (2) assess time-dependent effects over 3–6 months; (3) identify sources of heterogeneity through meta-regression. |
| **METHODS** |  |  |  |
| Eligibility criteria | 5 | Specify the inclusion and exclusion criteria for the review and how studies were grouped for the syntheses. | Methods, section 2.3 'Data screening and extraction'; Table 1 (Inclusion and exclusion criteria): Inclusion—RCTs of Wuqinxi in patients with grade 1–2 hypertension; intervention duration ≥3 months; no severe cardiopulmonary/hepatic/renal dysfunction. Exclusion—non-Wuqinxi interventions, non-RCTs, SBP <130 or DBP <90 mmHg, severe comorbidities, duration <3 or >12 months, incomplete data. |
| Information sources | 6 | Specify all databases, registers, websites, organisations, reference lists and other sources searched or consulted to identify studies. Specify the date when each source was last searched or consulted. | Methods, section 2.2 'Literature search': Four electronic databases searched—CNKI, PubMed, Web of Science Core Collection, and Embase; search period January 2000 to June 30, 2026; reference lists of included studies and relevant reviews hand-searched; no language restrictions. |
| Search strategy | 7 | Present the full search strategies for all databases, registers and websites, including any filters and limits used. | Methods, section 2.2 'Literature search': Search strategy combined subject headings and free-text terms using Boolean operators. Example: ("Five-Animal Exercise" OR "Five Animal Frolics" OR "Wuqinxi" OR "Wu Qin Xi Qigong") AND ("hypertension" OR "high blood pressure" OR "systolic blood pressure" OR "diastolic blood pressure" OR "cardiovascular risk"). No language restrictions applied. |
| Selection process | 8 | Specify the methods used to decide whether a study met the inclusion criteria of the review, including how many reviewers screened each record and each report retrieved, whether they worked independently, and if applicable, details of automation tools used in the process. | Methods, section 2.3 'Data screening and extraction'; Results, section 3.1 'Study selection'; Figure 1: Titles/abstracts screened, followed by full-text review of potentially eligible studies. Disagreements resolved through discussion with a third researcher. PRISMA flow diagram (Figure 1) shows progression from 167 records to 11 included studies. |
| Data collection process | 9 | Specify the methods used to collect data from reports, including how many reviewers collected data from each report, whether they worked independently, any processes for obtaining or confirming data from study investigators, and if applicable, details of automation tools used in the process. | Methods, section 2.3 'Data screening and extraction': Data extracted using a predefined form covering (1) basic literature information (author, title, study type); (2) intervention type (Wuqinxi confirmation); (3) measurement indices (SBP, DBP, HDL, LDL). Two researchers independently evaluated study quality; discrepancies resolved with a third researcher. |
| Data items | 10a | List and define all outcomes for which data were sought. Specify whether all results that were compatible with each outcome domain in each study were sought (e.g., for all measures, time points, analyses), and if not, the methods used to decide which results to collect. | Methods, section 2.3 'Data screening and extraction'; section 2.5 'Statistical methods': Primary outcomes—SBP, DBP, HDL, LDL; secondary outcomes included triglycerides (TG) and resting heart rate where reported. All compatible post-intervention measurements were extracted. |
| Data items | 10b | List and define all other variables for which data were sought (e.g., participant and intervention characteristics, funding sources). Describe any assumptions made about any missing or unclear information. | Methods, section 2.3 'Data screening and extraction'; Results, section 3.2 'Characteristics of included studies'; Table 2: Sample size (T/C), gender ratio, mean age, intervention/control details, intervention duration (months), sessions per week, baseline blood pressure, and outcome indicators. |
| Study risk of bias assessment | 11 | Specify the methods used to assess risk of bias in the included studies, including details of the tool(s) used, how many reviewers assessed each study and whether they worked independently, and if applicable, details of automation tools used in the process. | Methods, section 2.4 'Quality assessment'; Results, section 3.3 'Risk of bias and quality assessment'; Figure 2; Table 3: Two tools used—(1) Cochrane Risk of Bias Tool (seven domains: random sequence generation, allocation concealment, blinding, incomplete outcome data, selective reporting, etc.); (2) PEDro scale (10 scored criteria, 0–10 points). Two researchers independently assessed quality; disagreements resolved by a third researcher. No automation tools used. |
| Effect measures | 12 | Specify for each outcome the effect measure(s) (e.g., risk ratio, mean difference) used in the synthesis or presentation of results. | Methods, section 2.5 'Statistical methods': Continuous outcomes pooled using standardized mean difference (SMD) with 95% CI to account for differing measurement scales; mean difference (MD) also calculated for lipid outcomes reported in consistent units (mmol/L). Analyses performed using RevMan 5.4.1. |
| Synthesis methods | 13a | Describe the processes used to decide which studies were eligible for each synthesis (e.g., tabulating the study intervention characteristics and comparing against the planned groups for each synthesis (item #5)). | Methods, section 2.5 'Statistical methods'; section 2.6 'Sensitivity analysis': Studies were eligible for synthesis if they met inclusion criteria and reported compatible outcome measures. Pre-specified sensitivity exclusions addressed methodological quality, control-group design, and outlier/small-sample issues. |
| Synthesis methods | 13b | Describe any methods required to prepare the data for presentation or synthesis, such as handling of missing summary statistics, or data conversions. | Methods, section 2.5 'Statistical methods': Means and standard deviations extracted; SMD used to standardize across measurement scales. Missing or inconsistent data addressed through pre-specified sensitivity exclusions rather than imputation. |
| Synthesis methods | 13c | Describe any methods used to tabulate or visually display results of individual studies and syntheses. | Methods, section 2.5 'Statistical methods'; Results, section 3.4 'Meta-analysis results'; Figures 3–10; Tables 2–4: Forest plots present individual and pooled effect estimates; Table 2 summarizes study characteristics; Table 3 presents PEDro scores; Table 4 presents GRADE ratings; Figure 1 is the PRISMA flow diagram; Figure 2 summarizes risk of bias. |
| Synthesis methods | 13d | Describe any methods used to synthesize results and provide a rationale for the choice(s). If meta-analysis was performed, describe the model(s), method(s) to identify the presence and extent of statistical heterogeneity, and software package(s) used. | Methods, section 2.5 'Statistical methods': Random-effects models (DerSimonian–Laird method) used when substantial heterogeneity was present (I² > 50%, P ≤ 0.1); fixed-effect models used when heterogeneity was low. Heterogeneity assessed using χ² test and I² statistic. All analyses conducted in RevMan 5.4.1. |
| Synthesis methods | 13e | Describe any methods used to explore possible causes of heterogeneity among study results (e.g., subgroup analysis, meta-regression). | Methods, section 2.5 'Statistical methods'; section 2.6 'Sensitivity analysis'; Results, sections 3.4.1.1 and 3.4.2.1: Univariate and multivariate mixed-effects meta-regression explored moderators including total sample size, intervention duration, sessions per week, baseline SBP/DBP, control type, and mean age. Subgroup analyses by intervention duration were also conducted (section 3.4.2.2). |
| Synthesis methods | 13f | Describe any sensitivity analyses conducted to assess robustness of the synthesized results. | Methods, section 2.6 'Sensitivity analysis'; Results, section 3.4: Pre-specified sensitivity analyses sequentially excluded studies with (1) critical methodological flaws, (2) control-group design violations of the add-on principle (e.g., acupuncture, health education controls), and (3) extreme effect sizes (\|SMD\| > 2.0) or very small samples (<40/arm). Three studies excluded (Lang Q 2024; Niu Y 2023; Zhou H 2022). Post-sensitivity: SBP SMD improved from −1.06 to −0.53, I² from 90% to 12%; DBP SMD from −1.16 to −0.67, I² from 90% to 46%. |
| Reporting bias assessment | 14 | Describe any methods used to assess risk of bias due to missing results in a synthesis (arising from reporting biases). | Methods, section 2.7 'Funnel plot analysis': Funnel plots were not generated for any outcome comparison because all syntheses included fewer than 10 studies, consistent with PRISMA 2020 guidance against interpreting funnel plot asymmetry in small meta-analyses. |
| Certainty assessment | 15 | Describe any methods used to assess certainty (or confidence) in the body of evidence for an outcome. | Results, section 3.4.6 'GRADE evidence quality assessment'; Table 4: GRADE framework used to rate evidence certainty for each outcome, with downgrading for risk of bias (≥25% studies at high risk), inconsistency (I² > 50% unexplained), and imprecision (small sample size or CI crossing clinical decision thresholds). |
| **RESULTS** |  |  |  |
| Study selection | 16a | Describe the results of the search and selection process, from the number of records identified in the search to the number of studies included in the review, ideally using a flow diagram. | Results, section 3.1 'Study selection'; Figure 1: From 167 records across four databases, 35 duplicates and 18 non-journal/non-thesis records removed; 114 screened by title/abstract; 81 excluded; 35 full texts assessed; 24 excluded (16 non-RCT/non-Wuqinxi, 8 invalid duration); 11 RCTs (770 participants) included. |
| Study selection | 16b | Cite studies that might appear to meet the inclusion criteria, but which were excluded, and explain why they were excluded. | Results, section 3.1 'Study selection'; Figure 1: Twenty-four full-text reports excluded with reasons—16 were non-RCTs or non-Wuqinxi interventions; 8 had intervention durations outside the eligible range (<3 or >12 months). |
| Study characteristics | 17 | Cite each included study and present its characteristics. | Results, section 3.2 'Characteristics of included studies'; Table 2: All 11 included studies cited with detailed characteristics—author/year, sample size (T/C), gender ratio, mean age, intervention/control type, duration (months), sessions per week, baseline BP, and outcome measures. Included studies: He J 2019; Yuan Z 2011; Lang Q 2024; Li W 2015; Niu Y 2023; Xin S 2016; Zhou H 2022; Zhu Y 2025; Lin H 2013; Ru L 2013; Sun H 2015. |
| Risk of bias in studies | 18 | Present assessments of risk of bias for each included study. | Results, section 3.3 'Risk of bias and quality assessment'; Figure 2; Table 3: Cochrane risk-of-bias summary presented in Figure 2; PEDro scores in Table 3 show all included studies scored 6/10 (moderate quality), with consistent deficiencies in concealed allocation and blinding of participants, therapists, and assessors. |
| Results of individual studies | 19 | For all outcomes, present, for each study: (a) summary statistics for each group (where appropriate) and (b) an effect estimate and its precision (e.g., confidence/credible interval), ideally using structured tables or plots. | Results, section 3.4 'Meta-analysis results'; Figures 3–10: Forest plots for SBP (Figures 3–4, before/after sensitivity), DBP (Figures 7–8), TG, HDL (Figure 9), and LDL (Figure 10) present individual-study means, SDs, SMDs with 95% CIs, and pooled estimates. |
| Results of syntheses | 20a | For each synthesis, briefly summarize the characteristics and risk of bias among contributing studies. | Results, section 3.4 'Meta-analysis results': Each outcome subsection reports the number of included RCTs and participants (e.g., 11 RCTs pre-sensitivity, 8 post-sensitivity for SBP/DBP, n=560; 3 RCTs, n=170 for HDL/LDL/TG), along with risk-of-bias context (all studies moderate quality, PEDro = 6). |
| Results of syntheses | 20b | Present results of all statistical syntheses conducted. If meta-analysis was done, present for each the summary estimate and its precision (e.g., confidence/credible interval) and measures of statistical heterogeneity. If comparing groups, describe the direction of the effect. | Results, sections 3.4.1–3.4.5: Post-sensitivity pooled estimates—SBP: SMD = −0.53 (95% CI: −0.72 to −0.35; I² = 12%); DBP: SMD = −0.67 (95% CI: −0.91 to −0.42; I² = 46%); HDL: SMD = 0.71 (95% CI: 0.40 to 1.02; I² = 0%); LDL: SMD = −0.69 (95% CI: −1.32 to −0.07; I² = 86%); TG: SMD = −0.28 (95% CI: −0.61 to 0.05; I² = 18%; not significant). All effects favor Wuqinxi except TG (non-significant). |
| Results of syntheses | 20c | Present results of all investigations of possible causes of heterogeneity among study results. | Results, sections 3.4.1.1, 3.4.2.1, 3.4.2.2, and 4.5: Multivariate meta-regression identified mean age as a significant moderator for SBP (β = 0.0734, P = 0.0019) and control type as significant (overall P = 0.0182; acupuncture β = −1.9830, P = 0.0168). For DBP, mean age showed a marginal effect (β = 0.0486, P = 0.0795). Subgroup analysis by intervention duration reported in section 3.4.2.2. |
| Results of syntheses | 20d | Present results of all sensitivity analyses conducted to assess the robustness of the synthesized results. | Results, sections 3.4.1 and 3.4.2: Sensitivity analyses excluding Lang Q (2024), Niu Y (2023), and Zhou H (2022) reduced SBP heterogeneity from I² = 90% to 12% and DBP from I² = 90% to 46%, with effect sizes attenuating but remaining statistically significant (SBP: −1.06 to −0.53; DBP: −1.16 to −0.67), confirming robustness. |
| Reporting biases | 21 | Present assessments of risk of bias due to missing results (arising from reporting biases) for each synthesis assessed. | Methods, section 2.7 'Funnel plot analysis': Reporting bias could not be formally assessed because all outcome syntheses included fewer than 10 studies; funnel plots were therefore not generated, per PRISMA 2020 guidance. |
| Certainty of evidence | 22 | Present assessments of certainty (or confidence) in the body of evidence for each outcome assessed. | Results, section 3.4.6 'GRADE evidence quality assessment'; Table 4: GRADE ratings—SBP: Moderate (downgraded for risk of bias); DBP: Moderate (downgraded for risk of bias); HDL: Low (downgraded for risk of bias and imprecision); LDL: Very low (downgraded for risk of bias, inconsistency, and imprecision). |
| **DISCUSSION** |  |  |  |
| Discussion | 23a | Provide a general interpretation of the results in the context of other evidence. | Discussion, sections 4.1–4.4: Interprets pooled BP and lipid outcomes in context of other evidence; includes direct comparison with Baduanjin (section 4.2; Table 5), discussion of plausible biological mechanisms (section 4.3), and statistical/clinical interpretation including absolute BP reductions of 5.3 mmHg SBP and 6.7 mmHg DBP (section 4.4). |
| Discussion | 23b | Discuss any limitations of the evidence included in the review. | Discussion, section 4.6.2 'Limitations': Notes moderate overall trial quality (all PEDro = 6), consistent lack of allocation concealment and blinding, single-country evidence base (all studies from China), small sample sizes for lipid outcomes, and high heterogeneity for LDL. |
| Discussion | 23c | Discuss any limitations of the review processes used. | Discussion, section 4.6.2 'Limitations': Acknowledges limited long-term follow-up (3–6 months), potential co-intervention confounding, inability to assess publication bias (fewer than 10 studies per outcome), and restricted meta-regression power due to the small number of trials. |
| Discussion | 23d | Discuss implications of the results for practice, policy, and future research. | Discussion, sections 4.7 'Implications for clinical practice and public health policy' and 4.8 'Conclusion': Recommends Wuqinxi as an adjunctive non-pharmacological intervention (5–6 sessions/week over 3–6 months), particularly for younger middle-aged patients; calls for future multicenter RCTs with standardized protocols, larger samples, and longer follow-up. |
| **OTHER INFORMATION** |  |  |  |
| Registration and protocol | 24a | Provide registration information for the review, including register name and registration number, or state that the review was not registered. | Methods, section 2.1 'Study design': Protocol prospectively registered with PROSPERO prior to data extraction; registration number CRD420261396941. |
| Registration and protocol | 24b | Indicate where the review protocol can be accessed, or state that a protocol was not prepared. | Methods, section 2.1 'Study design': Protocol available at https://www.crd.york.ac.uk/PROSPERO/view/CRD420261396941 |
| Registration and protocol | 24c | Describe and explain any amendments to information provided at registration or in the protocol. | Methods, section 2.1 'Study design': The review was conducted in accordance with the registered protocol; no amendments are described. |
| Support | 25 | Describe sources of financial or non-financial support for the review, and the role of the funders or sponsors in the review. | Declarations, Funding: "This research received no specific grant from any funding agency in the public, commercial, or not-for-profit sectors." |
| Competing interests | 26 | Declare any competing interests of review authors. | Declarations, Competing interests: "The authors declare that they have no competing interests." |
| Availability of data, code and other materials | 27 | Report which of the following are publicly available and where they can be found: template data collection forms; data extracted from included studies; data used for all analyses; analytic code; any other materials used in the review. | Declarations, Availability of data and materials: "All data generated or analysed during this study are included in this published article and its supplementary information files. The datasets used are from previously published studies listed in the reference section." The PRISMA 2020 checklist is provided as Supplementary Material 1. |
